# Single organelle analysis to characterize mitochondrial function and crosstalk during viral infection

**DOI:** 10.1038/s41598-019-44922-9

**Published:** 2019-06-11

**Authors:** Annika Schneider, Sandra Kurz, Katrin Manske, Marianne Janas, Mathias Heikenwälder, Thomas Misgeld, Michaela Aichler, Sebastian Felix Weissmann, Hans Zischka, Percy Knolle, Dirk Wohlleber

**Affiliations:** 1 Institute of Molecular Immunology and Experimental Oncology, Klinikum rechts der Isar, Technical University of Munich, Munich, 81567 Germany; 2 Division of Chronic Inflammation and Cancer, German Cancer Research Center (DKFZ), Heidelberg, 69120 Germany; 3 Institute of Neuronal Cell Biology, Technical University of Munich, Munich, Germany; German Center for Neurodegenerative Diseases, Munich, 80802 Germany; 4 Research Unit Analytical Pathology, Helmholtz Zentrum München, Ingolstädter Landstraße 1, Oberschleißheim, 85764 Germany; 5 Sony Biotechnology, Sony Europe Ltd., Weybridge, Surrey KT13 0XW UK; 6 Institute of Molecular Toxicology and Pharmacology, Helmholtz Center Munich, German Research Center for Environmental Health, Neuherberg, 85764 Germany; 7 Institute of Toxicology and Environmental Hygiene, Technical University of Munich, Munich, 80802 Germany

**Keywords:** Flow cytometry, Viral infection

## Abstract

Mitochondria are key for cellular metabolism and signalling processes during viral infection. We report a methodology to analyse mitochondrial properties at the single-organelle level during viral infection using a recombinant adenovirus coding for a mitochondrial tracer protein for tagging and detection by multispectral flow cytometry. Resolution at the level of tagged individual mitochondria revealed changes in mitochondrial size, membrane potential and displayed a fragile phenotype during viral infection of cells. Thus, single-organelle and multi-parameter resolution allows to explore altered energy metabolism and antiviral defence by tagged mitochondria selectively in virus-infected cells and will be instrumental to identify viral immune escape and to develop and monitor novel mitochondrial-targeted therapies.

## Introduction

Mitochondria are crucial for cellular energy metabolism, critically involved in the coordination of signalling processes within cells and orchestrate induction of apoptotic cell death^1,2^. Besides this, cell-autonomous defence mechanisms during viral infection link innate immune sensing of infection and inflammation at the level of mitochondria^3,4^. The research in the recent years has expanded our knowledge about the different roles of mitochondria. For the different functions mitochondrial shape and motility, but also size, are important and are highly dynamic processes^5^. Mitochondrial shape and size are continuously changed during the dynamics of mitochondrial fusion and fission and mitochondrial turnover is controlled by mitophagy^5^. Viruses modify the host cell to create an ideal ambience, which includes metabolic support for viral gene expression and replication. Such modifications of cellular metabolism and structure of viruses can also affect mitochondria. There are more and more reports about viruses known to influence mitochondrial dynamics. Viruses known to enhance mitochondrial fission are hepatitis B virus (HBV), hepatitis C virus (HCV) and Epstein-Barr virus^6–9^. Viruses, which interfere with or enhance mitophagy are HBV, HCV and measles virus^6–8,10^. SARS coronavirus is reported to enhance the fusion of mitochondria^11^. But the influence of viral infection on mitochondrial membrane potential and stress response has not been addressed in detail because of methodological constraints. So far, analysis of mitochondria and their functions relied mostly on bulk analysis of mitochondrial populations analysed *ex vivo*. In infected tissues where both, infected and non-infected cells are simultaneously present, it is very difficult to discriminate between mitochondria from infected versus healthy non-infected cells. This may be achieved by serial tissue sections analysed by electron microscopy, where viral particles could be visualized. However, this is a very time demanding process yielding results with little statistical power. We therefore aimed to develop a technology, where high numbers of single mitochondria and their function can be analysed in the context of viral infection in order to characterize changes induced by viral infection. We chose the liver, and more specifically hepatocytes, as model system, because they serve as natural targets for many viral infections *in vivo*^12^ and harbour a high number of mitochondria for analysis^13^.

## Results

### Purification of mitochondria from virus-infected livers

**Figure 1:**
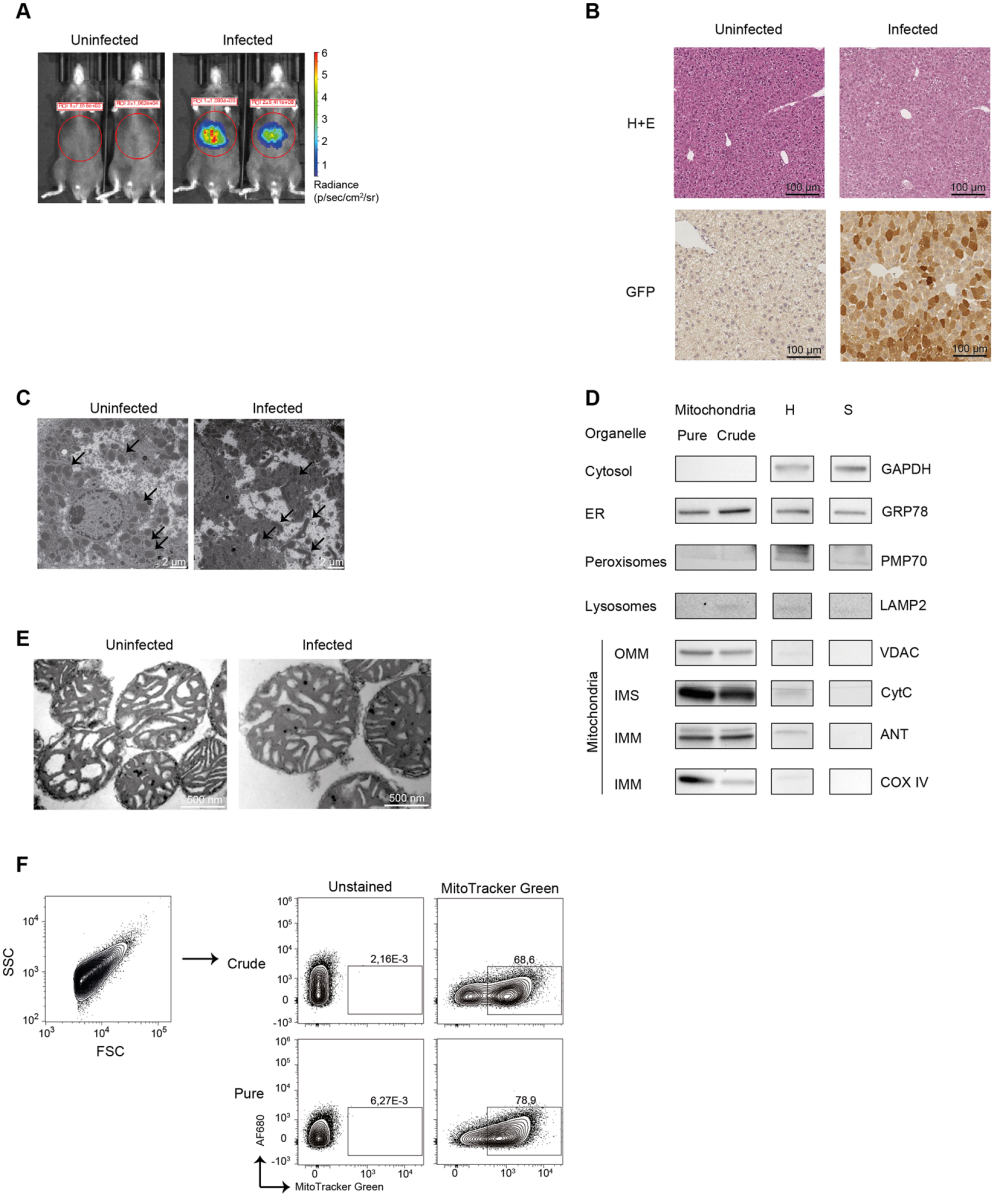
Viral infection changes mitochondrial size and membrane potential. (**A**) Bioluminescence imaging of mice two days after infection with Ad-CMV-GOL (5 × 10^8^ pfu/mouse). (**B**) H + E histochemical staining and GFP immunohistochemical staining (brown) of liver sections from mice infected two days before with Ad-CMV-GOL (5 × 10^8^ pfu/mouse). Scale bar = 100 µm. (**C**) Electron microscopy of liver sections from mice uninfected (left) and infected (right) with 5 × 10^8^ pfu/mouse Ad-CMV-GOL. Liver was removed two days after infection, fixed and subjected to electron microscopy. Arrows indicate mitochondria. Scale Bar = 2 µm. (**D**) Western blot analysis of mitochondria isolations (crude and after gradient purification (pure)). Detection of cellular proteins of distinct organelles, cytosol and compartments of isolated mitochondria compared to total liver homogenate (H) and supernatant of mitochondria pellet (S). Abbreviations: OMM = outer mitochondrial membrane; IMS = Intermembrane space; IMM = Inner mitochondrial membrane. GRP78, VDAC and Cyt C were detected on one Blot after cropping of the membrane. Lamp2 and GAPDH were detected on the same Blot after striping of first antibodies. PMP70, ANT and COX4 were detected together on a separate cropped blot. Exposure times are as follows: GAPDH 10 s; GRP78 95 s; Cyt C, ANT, and COX4 180 s; VDAC 1115 s; LAMP2 1200 s. (**E**) Electron microscopy of purified mitochondria isolated from healthy and Ad-CMV-GOL livers as in (C) Scale bar = 500 nm. (**F**) Flow cytometric analysis of isolated mitochondria pre (crude) and post purification via gradient (pure) stained with Mitotracker Green FM (200 nM). Representative data from at least three independent experiments.

Adenoviruses can infect hepatocytes when reaching the systemic circulation and were used as hepatotropic viral vectors to deliver transgenes to hepatocytes. Infection with recombinant hepatotropic adenoviruses coding for molecular markers such as luciferase and GFP lead to liver/hepatocyte-targeted infection, as determined by *in vivo* bioluminescence measurement of luciferase activity and detection of GFP in around 50% of hepatocytes by liver immunohistochemistry (Fig. 1A,B and^14^). Studying mitochondrial morphological characteristics in hepatocytes by ultrastructural analysis with transmission electron microscopy proved difficult, because it would require analysis of serial sections and simultaneous detection of infecting adenovirus, which is impossible as recombinant adenoviruses are replication-deficient and detection of few incoming virus particles per cell is very demanding (Fig. 1C). We therefore purified mitochondria from homogenized liver tissue by discontinuous percoll density gradient centrifugation, which yielded pure mitochondrial preparations as determined by immunoblot analysis (Fig. 1D and^15^). Ultrastructural analysis of these purified mitochondria by electron microscopy yielded heterogeneous results with respect to morphology and size (Fig. 1E) again with the uncertainty of whether mitochondria where derived from infected or non-infected cells. Flow cytometry is a methodology that can be employed to sensitively detect and analyse mitochondria with the advantage of quantitative characterization of large numbers of mitochondria^16–19^. This prompted us to use flow cytometry to determine directly *ex vivo* physical parameters of mitochondria at the level of single organelles. Purified mitochondria were reliable detectable by forward scatter and side scatter analysis in flow cytometry (Fig. 1F). Staining with MitoTracker Green confirmed that isolation from liver tissue by density-gradient centrifugation yielded highly pure mitochondria (Fig. 1F). We therefore conclude that flow cytometry is a suitable method to analyse mitochondrial properties after viral infection.

### Viral infection increases size and mitochondrial fragility of liver mitochondria

**Figure 2:**
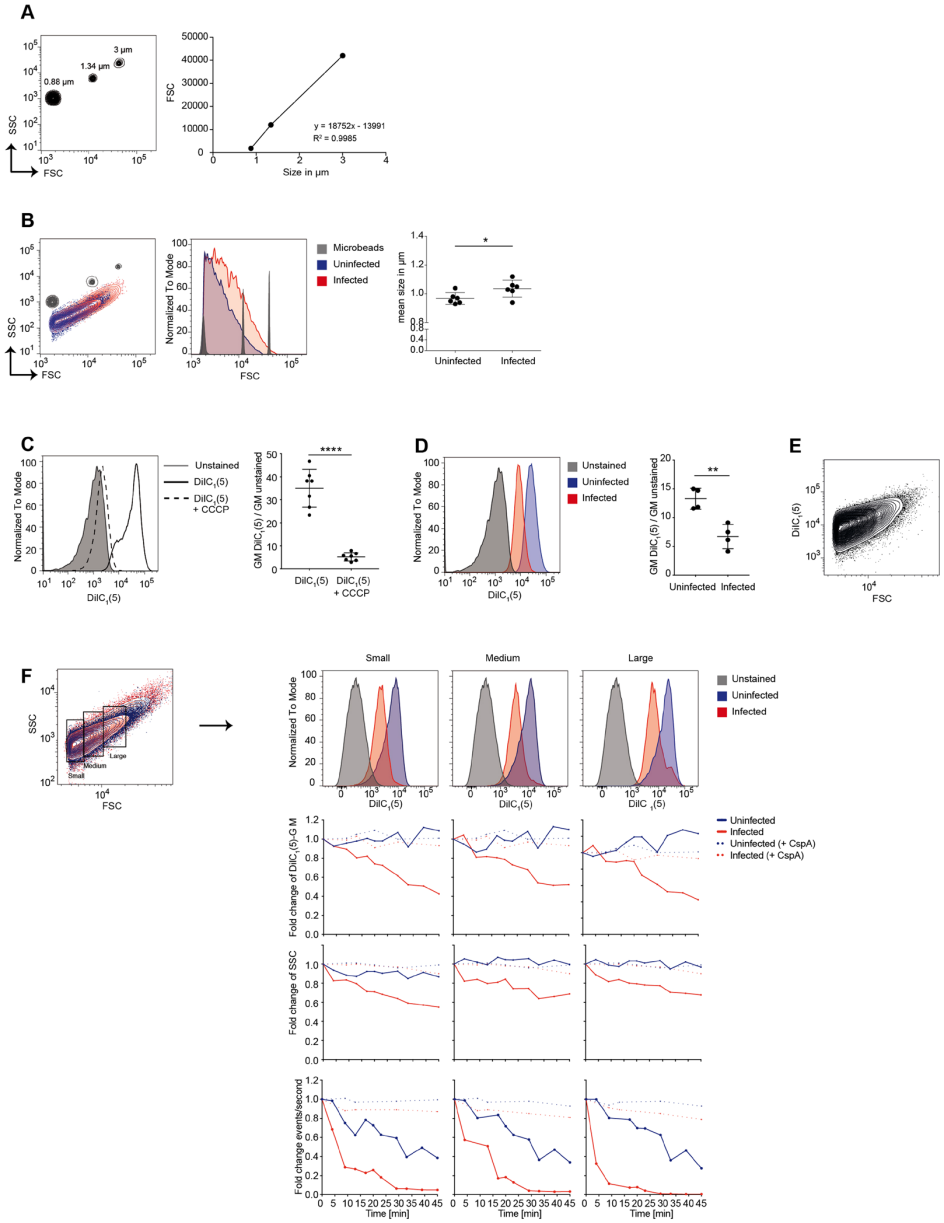
Specific determination of size, membrane potential and stress response of mitochondria from infected hepatocytes. (**A**) Flow cytometric analysis of size beads (0.88, 1.34 and 3 µm) from sperotech. Geometric mean (FSC) is plotted against corresponding size to calculate linear equation. (**B**) Flow cytometric analysis and quantification of purified mitochondria isolated from uninfected and Ad-CMV-GOL-infected mice (5 × 10^8^ pfu/mouse) mixed with sperotech size beads. Mean size in µm was calculated using linear equation calculated before (**A**). Six independent experiments were used for statistical analysis. (**C**) Flow cytometric analysis and quantification of mitochondrial membrane potential of isolated liver mitochondria using the membrane-potential dependent dye DilC_1_(5). Breakdown of mitochondrial membrane potential induced by CCCP (5 µM) observed by loss of DilC_1_(5) staining. Seven independent experiments were used for statistical analysis. (**D**) Flow cytometric analysis and quantification of the membrane potential by DilC_1_(5) staining of purified mitochondria from uninfected and Ad-CMV-GOL infected livers (as in (**B**)). Four independent experiments were used for statistical analysis. (**E**) Flow cytometric analysis of purified liver mitochondria for the membrane potential dye DilC_1_(5) (100 nM) against FSC. (**F**) Flow cytometric analysis at indicated time points after challenge with calcium of purified mitochondria isolated from uninfected and Ad-CMV-GOL-infected livers (as in (**B**)). Mitochondria were stained with DilC_1_(5) and challenged with 100 µM calcium with or without Cyclosporin A (5 µM); fold change in DilC1(5) geo-mean, SSC and events/second were plotted against time. Unless stated otherwise, data are representative from at least three independent experiments.

We first aimed to determine the influence of viral infection of the liver on the size of liver mitochondria by flow cytometry. To that end, we established a reference curve using polystyrene microparticles with defined sizes (0.88 µm, 1,34 µm and 3 µm). Forward scatter analysis of these polystyrene microparticles revealed clear demarcation of the differently sized microparticles and a direct linear correlation of forward scatter results with microparticle size (r^2^ = 0.99) (Fig. 2A), consistent with earlier reports that forward scatter measurements directly correlate with microparticle size down to 0.5 µm^20,21^. The flow cytometric analysis revealed that mitochondria isolated from healthy non-infected liver ranged in size from 0.8 µm up to 1.4 µm (Fig. 2B) assuming that isolated mitochondria are spherical in morphology, which is indicated by electron microscopy (see Fig. 1B). Since mitochondria from hepatocytes are much larger than those from non-parenchymal liver cells or immune cells, we assume that mitochondria ≥0.8 µm in size are derived from hepatocytes. Mitochondria purified from virus-infected livers had a slightly higher mean size compared to healthy liver (1.04 ± 0.06 µm compared to 0.97 ± 0.04 µm, respectively) and ranged in size from 0.8 µm up to 3 µm (Fig. 2B). Infection with recombinant replication-deficient adenoviruses is a well-established preclinical model system to study hepatotropic infections^22–24^. However, to confirm the results we repeated the experiments by infection with wildtype replication-competent lymphocytic choriomeningitis virus (LCMV). Also after LCMV-infection, we detected an increase of mitochondrial size confirming the results obtained after adenoviral infection (Supp. Fig. 1A). In order to investigate whether innate immunity generated during viral infection, was responsible for this increase in mitochondrial size, we induced a type I interferon response by application of poly I:C^25^. Flow cytometric analysis of mitochondria isolated after poly I:C application did not reveal any differences in their size compared to the control groups suggesting other mechanisms (Supp. Fig. 1B). The exact determination of the size of single mitochondria now opened the possibility to use this information for further analysis. Next, we evaluated mitochondrial functionality by determining the mitochondrial membrane potential using the potentiometric DilC_1_(5) fluorescence dye. Dose titration experiments of the DilC_1_(5) dye demonstrated a dose-dependent increase in fluorescence intensity in purified mitochondria (Supp. Fig. 1C). Upon addition of the electron chain uncoupling agent CCCP, we found a profound reduction in DilC_1_(5) fluorescence (Fig. 2C) demonstrating that flow cytometric determination of changes in DilC_1_(5) fluorescence reflected mitochondrial membrane potential. By flow cytometric analysis we observed a significant decrease in the mitochondrial membrane potential of mitochondria isolated from virus-infected vs. healthy livers after either adenoviral or LCMV infection compared to healthy controls (Fig. 2D and Supp. Fig. 1D). In contrast, we did not detect changes in the membrane potential after innate immune stimulation by poly I:C (Supp. Fig. 1E). Yet, the size of mitochondria may influence DilC_1_(5) signal intensity. Indeed, we found a direct correlation between mitochondrial size and DilC_1_(5) staining (Fig. 2E and Supp. Fig. 1F) suggesting that larger mitochondria purified from virus-infected livers should show higher DilC_1_(5) fluorescence intensity. We therefore compared mitochondria with the same size isolated from healthy or virus-infected livers. Such direct comparison demonstrated that mitochondria of the same size from healthy vs. virus-infected livers showed a remarkable decrease in the membrane potential of mitochondria from infected livers (Fig. 2F) and suggested that viral infection caused changes in mitochondrial functionality. Mitochondria also function to take up calcium from the cytosol and thereby coordinate cellular function^26^, which can also serve as a stress test. When challenged with high concentrations of calcium (100 µM), mitochondria isolated from virus-infected livers are much more fragile shown by time-dependent loss of membrane potential and change of their morphology indicated by decrease in side-scatter (Fig. 2F). This accurately detects mitochondrial swelling after loss of membrane potential following Ca^2+^ challenge which is also detected by bulk analysis with a classical stress test by adding Ca^2+^ and detection of loss of membrane potential by Rh123-fluorescence and swelling by measuring optical density at 540 nm (Supp. Fig. 1G)^18^. Consistent with the loss of membrane potential and changes in side-scatter signals, we detected loss of mitochondrial integrity after calcium challenge. Number of viable mitochondria detected per second by flow-cytometry declined after calcium challenge, consistent with loss of mitochondrial integrity, and did so much faster in samples from virus-infected livers (Fig. 2F). Comparing mitochondria with different sizes, it became evident that larger mitochondria are more fragile and disappeared more rapidly after Ca^2+^-challenge (Fig. 2F). Taken together, here we detected an increase in size and a decrease in membrane potential as well as mitochondrial fragility of liver mitochondria after viral infection. However, since both, non-infected as well as infected hepatocytes are present in livers after adenoviral infection (see Fig. 1B), current protocols for isolation and analysis yield a mixture of mitochondria derived from healthy as well as infected hepatocytes. This makes it necessary to develop a methodology, by which mitochondria from healthy and virus-infected hepatocytes can be separated in order to characterize changes in mitochondrial function specifically in virus-infected cells.

### Specific detection of mitochondria from virus-infected hepatocytes

**Figure 3:**
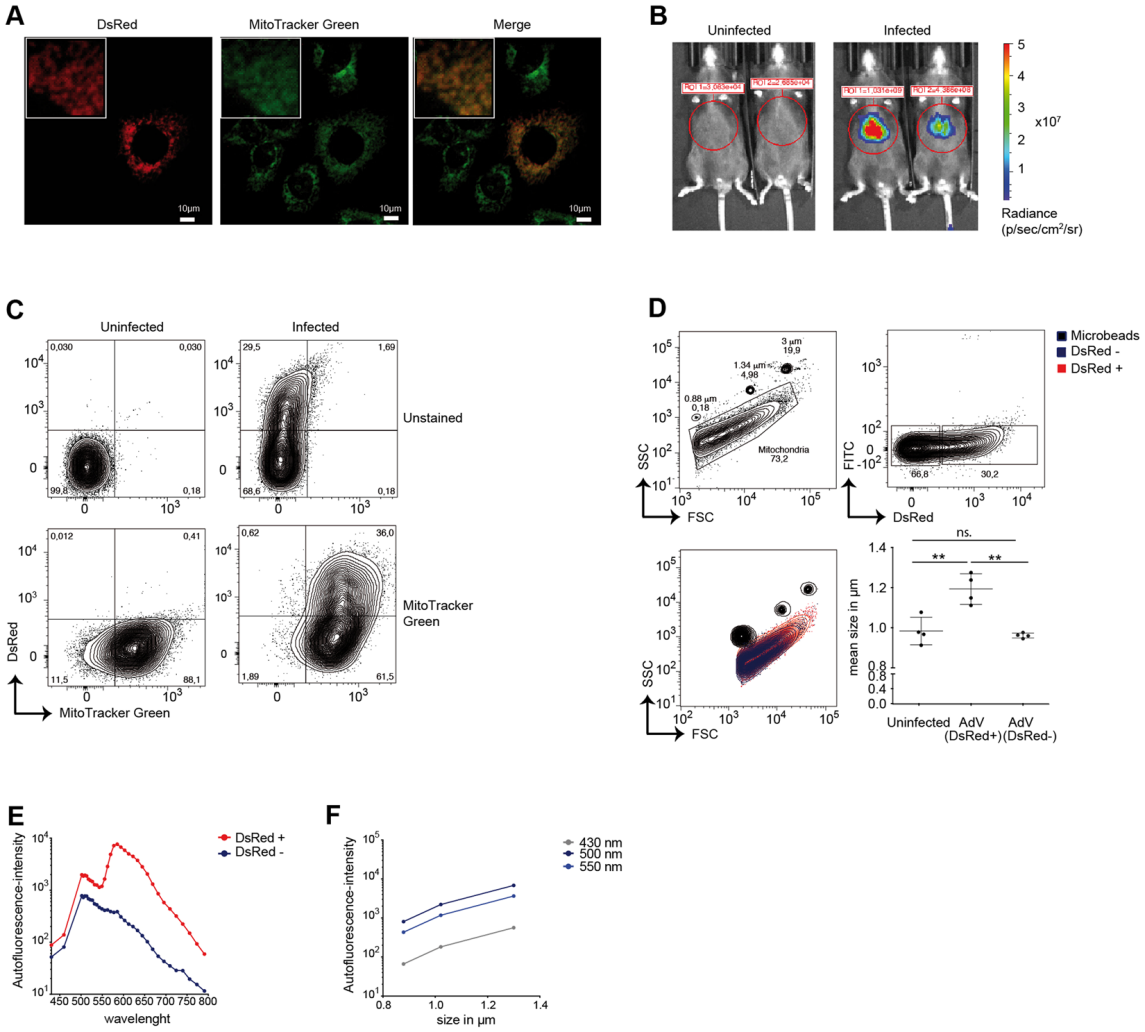
Specific detection of mitochondria from virus-infected hepatocytes. (**A**) Co-localization of DsRed and Mitotracker Green FM (200 nM) in AML-12 cells infected with Ad-CMV-mitoRL by MOI of 10. (**B**) Bioluminescence imaging of mice 2 days after infection with Ad-CMV-mitoRL (5 × 10^8^ pfu/mouse). (**C**) Flow cytometric analysis for DsRed and Mitotracker Green of purified mitochondria isolated from uninfected and Ad-CMV-mitoRL-infected mice (5 × 10^8^ pfu/mouse). (**D**) Flow cytometric analysis of purified mitochondria isolated from uninfected and Ad-CMV-mitoRL-infected mice (5 × 10^8^ pfu/mouse) mixed with spherotech size beads. Mitochondria from Ad-CMV-mitoRL-infected mice were gated on DsRed^+^ and DsRed^−^ events. Mean size in µm of isolated mitochondria was calculated using the linear equation calculated from size beads. (**E**) Flow cytometric analysis of autofluorescence signal from purified mitochondria of Ad-CMV-mitoRL-infected mice (5 × 10^8^ pfu/mouse). (**F**) Plot of autofluorescence signal against size of purified mitochondria from (**E**). Representative data from at least three independent experiments.

We generated a recombinant adenovirus expressing the fluorescent protein DsRed fused to a mitochondrial localization sequence (Ad-CMV-mitoRL) that accumulates and selectively labels mitochondria within infected cells (Supp. Fig. 2). We combined this mitochondrial labelling in infected cells with a multispectral flow cytometric single organelle measurement of isolated mitochondria. Upon infection of hepatocytes with Ad-CMV-mitoRL *in vitro* we detected mito-DsRed-fluorescence in mitochondria using confocal microscopy (Fig. 3A). Since Ad-CMV-mitoRL also codes for luciferase, we detected *in vivo* bioluminescence of the liver after infection, thus confirming successful infection of hepatocytes *in vivo* (Fig. 3B). This allowed us to test whether mitochondria from Ad-CMV-mitoRL-infected hepatocytes (mito-DsRed^+^ mitochondria) could be distinguished from those of non-infected hepatocytes (mito-DsRed^−^ mitochondria) within the same liver. After density-gradient purification, mitochondria isolated from virus-infected livers were counterstained with MitoTracker Green and analysed by flow cytometry allowing discrimination of mito-DsRed^+^ mitochondria from mito-DsRed^−^ mitochondria from the same liver (Fig. 3C). Mito-DsRed^+^ mitochondria from virus-infected hepatocytes had a mean size of 1.19 ± 0.06 µm as compared to mito-DsRed^−^ mitochondria from healthy hepatocytes with a mean size of 0.96 ± 0.01 µm (Fig. 3D). This confirmed the results obtained from mitochondria isolated from non-infected livers and further demonstrated a more pronounced size difference when mitochondria from virus-infected could be distinguished at the single organelle level from those of healthy hepatocytes. This was most likely related to a relative underestimation of size for mitochondria from virus-infected livers due to contamination with mitochondria from non-infected cells that are smaller than hepatocyte mitochondria. Yet, we cannot formally exclude that mito-DsRed localizing to mitochondria after infection with Ad-CMV-mitoRL may have contributed to the size difference. The almost identical forward scatter results and size of mito-DsRed^−^ mitochondria compared to mitochondria isolated from non-infected livers (Fig. 3D) indicated that there was no influence of viral infection in neighbouring hepatocytes on mitochondrial size after isolation. Differences in size of mitochondria may have also an influence on other parameters detected by flow cytometry and we therefore systematically measured mitochondrial autofluorescence from 450 to 800 nm using a spectral flow cytometer. As expected, we detected increased fluorescence at 590 nm in mito-DsRed^+^ mitochondria, where the maximum of DsRed fluorescence emission (590–650 nm) is expected^27^. Interestingly, we detected higher autofluorescence signals between 500 and 550 nm as well as above 650 nm in mito-DsRed^+^ mitochondria (Fig. 3E). As the strength of autofluorescence may be influenced by the size of mitochondria, we analysed autofluorescence signals against the size of isolated mitochondria (Fig. 3F). We found that autofluorescence intensity between 430 and 550 nm directly correlated with mitochondrial size, which may explain the higher autofluorescence observed in larger mito-DsRed^+^ mitochondria. Together, these data demonstrate the usefulness of single-organelle analysis by flow cytometry in combination with *in vivo* mitochondrial labelling in virus-infected hepatocytes to exactly determine physical parameters such as size or autofluorescence.

### Mitochondrial crosstalk enables changes in membrane potential

**Figure 4:**
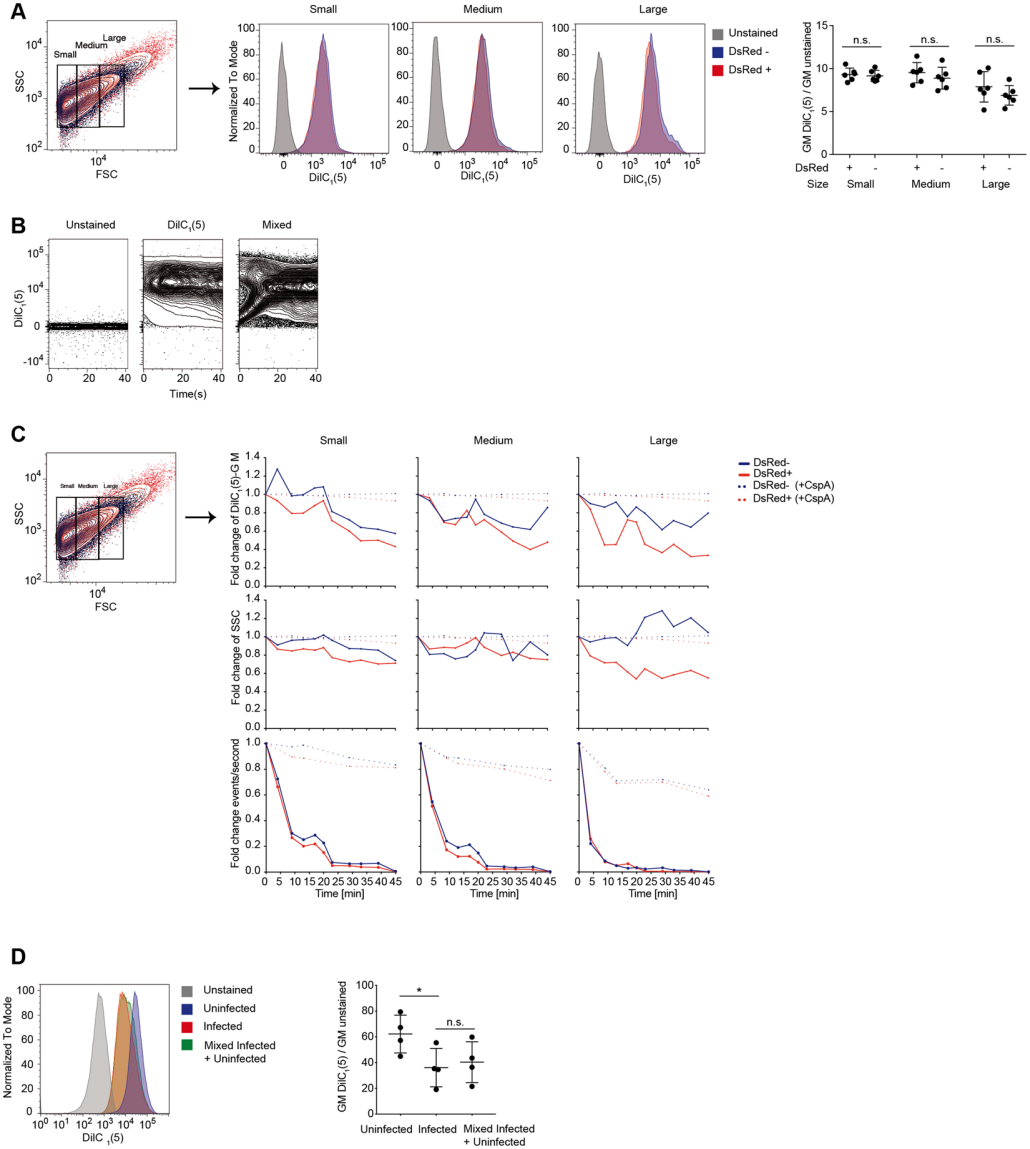
Mitochondrial cross-talk impacts on membrane potential. (**A**) Flow cytometric analysis and quantification of mitochondrial membrane potential by DilC_1_(5) (100 nM) of isolated mitochondria from Ad-CMV-mitoRL-infected mice (5 × 10^8^ pfu/mouse). Mitochondria were gated on mitochondria from infected cells (DsRed^+^) and mitochondria from uninfected cells (DsRed^−^). Membrane potential is plotted as staining intensity of DilC_1_(5) (100 nM). Six independent experiments were used for statistical analysis. (**B**) Flow cytometric analysis over time of DilC_1_(5) labelled mitochondria mixed with unstained mitochondria (measurement immediately after mixing both samples). (**C**) Flow cytometric analysis at indicated time points after challenge with calcium of purified DsRed- and DsRed + mitochondria isolated from Ad-CMV-mitoRL-infected livers (5 × 10^8^ pfu/mouse). Mitochondria were stained with DilC_1_(5) and challenged with 100 µM calcium with or without Cyclosporin A (5 µM); fold change in DilC1(5) geo-mean, SSC and events/second were plotted against time. (**D**) Flow cytometric analysis and quantification of membrane potential by DilC_1_(5) staining of mitochondria from uninfected livers with mitochondria from Ad-CMV-GOL infected livers at a ratio 1:1. Samples were mixed before staining procedure (measurement 1 h post staining). Four independent experiments were used for statistical analysis. Unless stated otherwise, data are representative from at least three independent experiments.

Since discrimination of mitochondria isolated from virus-infected compared to non-infected hepatocytes was reliably achieved using flow cytometry, we proceeded to test for changes in mitochondrial functionality upon infection. We assumed that the difference in membrane potential detected between mitochondria isolated from virus-infected livers compared to non-infected livers (see Fig. 2) has previously been underestimated, and that our method would allow to more specifically discriminate mitochondria from virus-infected hepatocytes compared to non-infected hepatocytes. We determined whether mito-DsRed^+^ differed from mito-DsRed^−^ mitochondria with respect to DilC_1_(5) fluorescence intensity. To our surprise, we found that the DilC_1_(5) signal was similar for all sizes of mito-DsRed^+^ compared to mito-DsRed^−^ mitochondria (Fig. 4A). Since DilC_1_(5) fluorescence was homogenous in all mitochondria isolated from Ad-CMV-GOL infected livers (see Fig. 1), although they consisted of a mixture of mitochondria from infected and non-infected hepatocytes, we wondered whether there was an exchange of molecules between mitochondria. Therefore, we mixed DilC_1_(5)-labelled mitochondria with non-labelled mitochondria and by time-dependent flow cytometric analysis found that DilC_1_(5) fluorescence decreased in pre-labelled and increased in un-labelled mitochondria reaching an equilibrium of intermediate fluorescence intensity within 30 seconds (Fig. 4B). However, mito-DsRed was not exchanged between mitochondria, because we found clearly distinct DsRed staining of mitochondria isolated from Ad-CMV-mitoRL-infected livers, and mito-DsRed^−^ mitochondria showed the same absent DsRed fluorescence intensity as mitochondria isolated from non-infected livers. In order to further evaluate mitochondrial functionality, we challenged mitochondria with Ca^2+^ as stress test and performed time kinetic measurements of DilC_1_(5) fluorescence and side-scatter of mito-DsRed^+^ and mito-DsRed^−^ mitochondria isolated from Ad-CMV-mitoRL infected livers. Remarkably, the differences in mitochondrial characteristics observed when comparing mitochondria isolated from infected livers to mitochondria from non-infected livers (see Fig. 2F) where not present any more when comparing mito-DsRed^+^ to mito-DsRed^−^ mitochondria originating from the same liver. In fact, loss of DilC_1_(5) fluorescence, decrease in side scatter and mitochondrial events were the same for mito-DsRed^+^ mitochondria as compared to mito-DsRed^−^ mitochondria (Fig. 4C). When in direct physical contact with mito-DsRed^+^ mitochondria, also mito-DsRed^−^ mitochondria showed the same fragility as mitochondria from virus-infected hepatocytes. There, was still a small difference in the large mitochondrial group after calcium stimulation and flow cytometric analysis of the SSC and DilC_1_(5) which could be explained by the fact that 5 to 10 minutes after calcium stimulation the number of events was drastically reduced. Only approximately 10% from the initial number of events are still detectable (shown by number of events/s). Because of the statistical variation the conclusions at later time points has to be taken with caution. Interestingly, also mixing of DilC_1_(5) labelled mitochondria isolated from either Ad-CMV-GOL- or LCMV-infected with those from healthy uninfected livers yielded in rapid loss of mitochondrial membrane potential to that measured in mitochondria from infected livers (Fig. 4D and Supp. Fig. 2) Taken together these data demonstrate that mitochondria which are in close physical proximity exchange information leading to changes in mitochondrial membrane potential but not in mitochondrial size.

## Discussion

Here, we describe the influence of viral infection on the phenotype and function of mitochondria employing a new methodology combining spectral flow cytometry with virus-encoded markers to simultaneously evaluate multiple mitochondrial parameters at the level of single organelles. Most studies involve confocal microscopy to detect mitochondria, which is also available in an automated manner to quantify large datasets of mitochondria^28^. While most of these microscopic studies are performed in cell cultures to explore mitochondrial dynamics at the level of single cells, there are only few reports specifically detecting tagged mitochondria in tissues for *ex vivo* or *in vivo* analysis^29,30^. Since *in vivo* microscopic analysis of mitochondria requires a complex experimental setup, is rather time consuming and does not allow for analysis of large numbers of mitochondria, we aimed to establish a methodology to evaluate mitochondria directly *ex vivo* following isolation from virus-infected tissue. So far, most of available methods analyse properties of mitochondria *ex vivo* at the level of mitochondrial populations, such as extracellular flux analysis, western blot analysis, calcium uptake or swelling assays. Beyond visualization by microscopy, flow cytometry has emerged as technology to characterize mitochondria^18,31,32^. However, mitochondria isolated from virus-infected tissues can be derived from both, virus-infected cells as well as healthy cells, which may skew the experimental results.

We therefore generated recombinant adenoviruses containing a mito-DsRed expression cassette to selectively label mitochondria of infected cells. Fusion of a fluorescent marker to mitochondrial target sequences has previously been reported to reliably and specifically label mitochondria as shown by confocal microscopy^33,34^. We combined virus-encoded mito-DsRed labelling of mitochondria to separate mitochondria of virus-infected cells from those originating from healthy cells, with the power of multi-parameter analysis by spectral flow cytometry. Using this methodology, we provide evidence that mitochondria can be reliably separated from virus-infected cells and that viral infection led to an increase in size as well as a decrease of mitochondrial membrane potential. Such changes in biophysical and functional properties of mitochondria were not triggered by innate immunity following recognition of infection through microbe-associated pattern recognition receptors indicating other reasons for these changes, which still have to be defined. Time kinetic measurements of single mitochondria by flow cytometry further allowed us to detect a previously unknown mitochondrial cross-talk that involves rapid exchange of small molecules like the potentiometric dye DilC_1_(5). Such exchange of molecules among mitochondria required physical contact, occurred within seconds and did not include mitochondrial matrix-embedded proteins. This indicates a dynamic regulation of mitochondrial properties by cell autonomous mechanisms that require further investigation.

Taken together, the combination of mitochondrial labelling through mito-DsRed together with single organelle analysis using spectral flow cytometry is ideally suited to further unravel biophysical and functional properties of mitochondria as well as mechanisms and consequences of mitochondrial interconnectivity in virus-infected cells. Given the important role of mitochondria in cellular metabolism, anti-viral defence, cell signalling and cell death, the multiparametric analysis of single mitochondria opens new avenues to explore these complex mitochondrial functions in more detail in virus-infected cells.

## Methods

### Mice

C57Bl/6 J mice were purchased from Charles River (Sulzfeld, Germany). Mice were maintained under specific pathogen-free (SPF) conditions in the central animal facility of the Klinikum rechts der Isar, in accordance with the guidelines of the Federation of Laboratory Animal Science Association. Animal experiments were approved by the Animal Care Commission of Bavaria. Male mice between the ages of 6–10 weeks were used.

### Generation of recombinant Ad-CMV-mitoRL

The expression cassette for cloning into recombinant adenovirus consists of the genes for the fluorescent protein DsRed linked to a mitochondrial targeting sequence and CBG99-luciferase separated by P2A linker sites from the Porcine Teschovirus 1 followed by a bGH poly(A) signal. Gene expression was driven by the ubiquitous minimal CMV-promoter (Ad-CMV-mitoRL). Ad-CMV-GOL generation has been reported before^23^.

Recombinant second generation serotype 5 adenoviruses were generated using the Gateway® technology from ThermoFisher as described before^23^. Briefly, expression cassettes with CMV promotor, DsRed linked to the mitochondrial targeting site, CBG99-luciferase and the bGH poly(A) signal were synthesized (Eurofins Genomics, Germany) and cloned into Gateway® pENTR™11 Dual Selection Vector (ThermoFisher Scientific, Germany). Recombination of pENTR™ with expression cassette into pAD/PL-DEST™ Gateway® Vector (ThermoFisher Scientific, Germany) was performed *in vitro* via the LR Clonase® Enzyme Mix (ThermoFisher Scientific, Germany). The obtained pAD/PL-DEST™ with expression cassette was linearized using the PacI restriction enzyme and the resulting adenoviral DNA was transfected with Lipofectamine 2000 (ThermoFisher Scientific, Germany) into HEK293 cells (CRL-1573™; ATCC, USA). Cell debris and supernatant were harvested when complete detachment of the cells occurred. This suspension was freeze/thawed, centrifuged and used for further infection of HEK293 cells. Cells from several cell culture dishes were harvested and resuspended in Tris-buffer and freeze/thawed three times. Cell debris was removed by centrifugation and supernatant purified by a two-step CsCl gradient ultracentrifugation. The band containing adenovirus was harvested and dialyzed. Virus titer was determined via adenovirus hexon titration. HEK293 cells were infected with serial dilutions of purified adenovirus. After 35 to 40 hours, cells were fixed with methanol, and virus infected cells were stained with anti-Hexon antibody (anti-Hexon 2297HRP, Acris, Germany) and detected via DAB (Dako, USA). The infected cells were counted and the titer was calculated.

### Bioluminescence imaging

Imaging of luciferase expression in infected mice was monitored by IVIS Lumina LT-Series III instrument (PerkinElmer LAS, Germany). Five minutes before measurement mice have been anesthetized with 2.5% Isofluran and treated intraperitoneally with 100 mg/kg bodyweight D-Luciferin-K-Salt (PJK GmbH, Germany).

### AML-12 cell line

AML-12 cells were seeded onto CollagenR-coated coverslips in a 24-well format in a density of 50000 cells/well in 500 µl Growth medium. 24 h post seeding growth medium was exchanged to infection medium and cells were infected with Ad-CMV-mitoRL at an MOI of 10. After 24 h of incubation cells were washed with prewarmed PBS and stained with 200 nM Mitotracker Green FM (ThermoFisher Scientific, Germany) in medium without supplements. After 30 minutes of incubation at 37 °C, media was replaced and cells were analysed using ZOE™ Fluorescent Cell Imager (BioRad, USA).

### Isolation of mitochondria from murine liver tissue

Heparin/NaCl (300 U/150 µl) was injected i.p. into the mouse 5 minutes prior to preparation. Mice were sacrificed and livers were perfused via portal vein for 1 minute with PBS to remove blood. Liver was removed and weighed, and the liver was rinsed with Isolation Buffer (220 mM Mannitol, 80 mM Sucrose, 10 mM HEPES, 1 mM EDTA, pH 7.4). The whole isolation procedure was performed on ice and in ice-cold Isolation Buffer. The tissue was rinsed with 1 ml Isolation Buffer and cut with a blunt end scissor into small pieces. The liver fragments were resuspended in 1 ml Isolation Buffer supplemented with 0.5% BSA and protease inhibitor (Protease inhibitor cocktail, EDTA-free, Roche, Switzerland) per 0.1 gram of weighted organ and homogenized in a Potter-Elvehjem with 3 strokes at 800 rpm. The homogenate was transferred to cooled 50 ml falcon and centrifuged at 600 x g for 10 minutes to remove nuclei, intact cells and cellular debris. The supernatant was transferred to a glass tube and centrifuged at 4000 x g for 10 minutes to sediment mitochondria. The received crude pellet was gently dislodged with a glass pestle from the side of the glass tube.

### Mitochondrial purification by density gradient centrifugation

Mitochondria were purified as previously described^35,36^. In brief, a discontinuous percoll density gradient was used for mitochondrial purification. Crude mitochondria were resuspended in IP-buffer (300 mM Sucrose, 5 mM TES, 0.2 mM EGTA, pH 6.9), loaded on a percoll density gradient (60%, 30% and 18% diluted in IPP Buffer: 300 mM Sucrose, 10 mM TES, 0.2 mM EGTA, 0.1% w/v BSA, pH 7.2) and separated at 9000 × g for 10 minutes. The phase containing mitochondria between 60% and 30% Percoll-layer was recovered with a glass pipette and transferred to a 30 ml glass tube, resuspended in 15 ml IP-buffer and centrifuged for further 10 minutes at 9000 × g. The pellet was washed again in 10 ml IP-buffer and centrifuged at 9000 × g for 10 minutes to get rid of remaining Percoll. The supernatant was removed and mitochondrial pellet was dislodged from the side of the glass tube. The received mitochondria were resuspended in 100 µl IP-Buffer and kept on ice.

### Determination of protein concentration

The protein content in the mitochondrial preparations was determined using the DC^TM^ Protein Assay kit (Bio Rad Laboratories, Germany). The Assay was performed according to the manufacturer´s protocol. Four different BSA-dilutions reaching from 0.25 mg/ml to 1.5 mg/ml in IP-buffer were used as standards. The optical density was measured at 750 nm with a Multiplate Reader (InfiniteM100 Pro, Tecan, Germany).

### Determining mitochondria by flow cytometry

Mitochondria were diluted to 10 µg protein per µl in ice-cold mitochondrial staining buffer MSB (0.2 M Saccharose, 10 mM MOPS-Tris, 5 mM Succinate, 1 mM phosphoric acid, 10 µM EGTA). The different mitochondrial probes were diluted in MSB, mixed with the mitochondrial dilution in a 1:1 ratio and incubated at room temperature for 20 minutes. The cell permeable carbocyanine-based Mitotracker Green probe (MTG, 200 nM), which contains a mildly thiol-reactive chloromethyl moiety, was used to selectively stain all undamaged mitochondria regardless of the membrane potential. DilC1(5) (100 nM), a cationic carbocyanine dye, was used to measure the membrane potential of isolated mitochondria. Mitochondria were pelleted at 9000 x g for 2 minutes and washed once in ice cold PBS. Mitochondrial pellet was resuspended in MSB to a final concentration of 10 µg/µl and stored on ice for analysis. Immediately before analysis, samples were diluted in ice-cold and filtered PBS to the final analysis concentration of 0.05 µg/µl. Samples were analysed using the Spectral Cell Analyzer SP6800 (Sony Biotechnology Inc, Japan). The sample flow rate was set to record about 1500 events per second. As mitochondrial uncoupling by the protonophore CCCP is well known to dissipate mitochondrial membrane potential (MMP), 5 µM CCCP (Sigma-Aldrich, St. Louis, Missouri, USA) was used as a positive control for membrane potential dependence of DiIC_1_(5) (Biotium, Hayward, USA). The mitochondrial permeability transition (MPT), a process characterized by a large increase of permeability of the inner mitochondrial membrane (IMM), leading to an influx of solutes with a molecular weight less than 1.5 kDa and water into the mitochondrion, is a Ca^2+^-induced process. The influx of solutes and water leads to swelling of mitochondria. In MPT-measurements 100 µM Ca^2+^ in MSB was added to induce swelling and samples were analyzed immediately after administration and every following 5 minutes for 45 minutes in total. CyclosporinA (Sigma-Aldrich, St. Louis, Missouri, USA) inhibiting MPT and thereby reversing the effect of Ca^2+^, was added at a concentration of 5 µM.

Mitochondrial size was determined using Polystyrene Particle Size Standard beads (Flow Cytometry Grade, spherotech) in three sizes: 0.88 μm, 1.34 μM and 3 μM. Beads of each size were separated via ultrasound, vortexed and 20000 beads/size were added per mL filtered PBS. Immediately before analysis, mitochondria were diluted in bead mixture to the final analysis concentration of 0,05 µg/mL. Data were analysed using FlowJo software (Version 10, FlowJo, Oregon, USA).

### Mitochondrial Swelling

Mitochondria were diluted in swelling buffer (SWP: 200 mM Sucrose, 10 mM MOPS-Tris, 5 mM Succinate, 1 mM Phosphate, 10 µM EGTA, 2 µM Rotenone) to a concentration of 0.75 µg/µl. 125 nM Rh123 was added to detect indirectly the mitochondrial membrane potential. 100 µM Calcium is added and the changes in mitochondria ultrastructure are detected via absorbance at 540 nm at 37 °C in parallel to Rh123 fluorescence emission (ex 505/em 535).

### Western-Blot

30 µg of protein per sample was loaded onto 4–20% Mini-PROTEAN® TGX Stain-Free™ Precast Gels (Bio Rad Laboratories, München) and separation was performed within a gel chamber filled with 1x SDS electrophoresis buffer at 100 V for 1 to 2 hours. After separation, proteins were blotted using the Trans-Blot® Turbo™ Mini PVDF Transfer Packs (Bio Rad Laboratories, Germany). Proteins were transferred onto membranes at 2.5 A for 30 minutes using the Trans-Blot Turbo™ (Bio Rad Laboratories, Germany). Membranes were blocked with 10% milk in TBS-T (TBS + 0.1% Tween-20) for 1 hour at room temperature, washed three times with TBS-T and incubated with primary antibodies in 5% BSA in TBS-T overnight at 4 °C. The membranes were washed three times with TBS-T and incubated for 4 hours at room temperature with HRP-coupled secondary antibodies in 10% milk powder in TBS-T. Blots were washed three times and developed using Cheluminate-HRP PicoDetect (Applichem GmbH, Germany), which was evenly distributed on the membrane. The luminescence was detected for up to 20 minutes using the Imaging-System ChemiDoc^TM^ XRS (Bio Rad Laboratories, Germany). To visualize several proteins on the same blot, primary and secondary antibodies were removed by incubating membranes for 45 minutes at 50 °C in Stripping buffer containing ß-mercaptoethanol. Subsequently membranes were washed three times with TBS-T and incubated as previously described with primary and secondary antibodies.

Following primary antibodies were used: Adenine nucleotide translocator (ANT) (Santa Cruz Biotechnology USA), Cytochrome-C-oxidase (COX IV), Cytochrome-C (Cyt-C), Glyceraldehyde 3-phosphate dehydrogenase (GAPDH), Glucose-regulated-protein 78 (GRP78), Histon 2B (H2B), Voltage dependent anion channel (VDAC) (all Cell Signaling Technology, USA), Lysosome-associated membrane protein 2 (LAMP2) (Thermo Fisher Scientific, USA), Peroxisomal membrane protein 70 (PMP70) (OriGene Technologies, USA), Following secondary antibodies were used: rabbit anti-goat HRP (Santa Cruz Biotechnology, USA), mouse anti-rabbit HRP, goat anti-mouse HRP (Jackson ImmunoResearch, UK).

### Histology

Mouse livers were fixed for 48 hours in 4% paraformaldehyde. Dehydrated livers (Leica ASP300S, Germany) were embedded in paraffin. Serial 2 µm-thin sections were prepared with a rotary microtome (HM355S, ThermoFisher Scientific, USA) and subjected to histological and immune-histochemical analysis. Hematoxylin-Eosin (HE) staining was performed on deparaffinized sections with Eosin and Mayer’s Haemalaun according to standard protocol.

Immunohistochemistry was performed using a BondMax RXm system (Leica, Wetzlar, Germany, all reagents from Leica) with primary antibody against eGFP (A-11122, diluted 1:500 in antibody diluent, Invitrogen, ThermoFisher Scientific, USA). Slides were deparaffinized, pre-treated with Epitope Retrieval solution 1 for 30 minutes. Bound antibody was detected with a Polymer Refine detection kit without post primary reagent and visualized with DAB as a dark brown precipitate. Counterstaining was done with hematoxyline.

### Electron microscopy

Tissues were fixed in 2.5% electron microscopy grade glutaraldehyde in 0.1 M sodium cacodylate buffer pH 7.4 (Science Services, Munich, Germany), postfixed in 2% aqueous osmium tetraoxide^37^, dehydrated in gradual ethanol (30–100%) and propylene oxide, embedded in Epon (Merck, Darmstadt, Germany) and cured for 48 hours at 60 °C. Semithin sections were cut and stained with toluidine blue. Ultrathin sections of 50 nm were collected onto 200 mesh copper grids, stained with uranyl acetate and lead citrate before examination by transmission electron microscopy (Zeiss Libra 120 Plus, Carl Zeiss NTS GmbH, Oberkochen, Germany). Pictures were acquired using a Slow Scan CCD-camera and iTEM software (Olympus Soft Imaging Solutions, Münster, Germany).

### Statistics

Student’s t tests were calculated using GraphPad Prism software. Significance was set at p < 0.05 and denoted as *p < 0.05, **p < 0.01, ***p < 0.001 and ***p < 0.0001. All results are expressed as the mean ± standard deviation (SD).

## Supplementary information

Supplementary Material

## Data Availability

The data within this manuscript are available from the corresponding author upon reasonable request.
