# Supplementary material for: Single organelle analysis to characterize mitochondrial function and crosstalk during viral infection
Source: Sci Rep. 2019 Jun 11;9:8492. doi: 10.1038/s41598-019-44922-9 (PMC6560178; doi:10.1038/s41598-019-44922-9)
Supplement: Supplementary file 1 — Supplementary Material [file 41598_2019_44922_MOESM1_ESM.pdf]

Single organelle analysis to characterize mitochondrial function and crosstalk during viral infection

Annika Schneider[1,8], Sandra Kurz[1,8], Katrin Manske[1], Marianne Janas[1], Matthias Heikenwälder[2], Thomas Misgeld[3], Michaela Aichler[4], Sebastian Felix Weissmann[5], Hans Zischka[6,7], Percy Knolle[1], Dirk Wohlleber[1,*]

[1] Institute of Molecular Immunology and Experimental Oncology, Klinikum rechts der Isar, Technical University of Munich, Munich, 81567, Germany

[2] Division of Chronic Inflammation and Cancer, German Cancer Research Center (DKFZ), Heidelberg, 69120, Germany.

[3] Institute of Neuronal Cell Biology, Technical University of Munich, Munich, Germany; German Center for Neurodegenerative Diseases, Munich, 80802, Germany

[4] Research Unit Analytical Pathology, Helmholtz Zentrum München, Ingolstädter Landstraße 1, Oberschleißheim, 85764, Germany

[5] Sony Biotechnology, Sony Europe Ltd., Weybridge, Surrey, KT13 0XW, UK

[6] Institute of Molecular Toxicology and Pharmacology, Helmholtz Center Munich, German Research Center for Environmental Health, Neuherberg, 85764, Germany

[7] Institute of Toxicology and Environmental Hygiene, Technical University of Munich, Munich, 80802, Germany

[8] These authors contributed equally

Supplemental Figure 1: Analysis of mitochondria from virus-infected livers.

(**A**) Flow cytometric analysis of purified mitochondria isolated from livers of uninfected and LCMV-infected mice (1 x 10^4 pfu / mouse) mixed with spherotech size beads. Mean size in µm was calculated using linear equation calculated from size beads by linear regression. (**B**) Flow cytometric analysis of purified mitochondria isolated from livers of untreated and poly I:C treated mice (200 µg / mouse) mixed with sperotech size beads. Mean size in µm was calculated using linear equation calculated from size beads by linear regression. (**C**) Flow cytometric analysis of isolated liver mitochondria. Titration of the membrane-potential dependent dye $DiLC_1(5)$ (50nM - 400nM). (**D**) Flow cytometric analysis of the membrane potential by $DilC_1(5)$ staining of purified mitochondria from uninfected and LCMV infected livers (as in (A)). (**E**) Flow cytometric analysis of the membrane potential by $DilC_1(5)$ staining of purified mitochondria from livers of untreated and poly I:C treated mice (as in (B)). (**F**) Flow cytometric analysis of purified liver mitochondria for the membrane potential dye $DilC_1(5)$ (100 nM) against FSC. Population was divided in subpopulation of different FCS ranges to plot MFI-FSC against MFI-$DilC_1(5)$ for linear regression. (**G**) At d2 after infection with Ad-CMV-GOL (5 x 10^8 PFU/mouse) mitochondria were isolated, challenged with calcium (100 µM) and mitochondrial membrane potential (Rh123 fluorescence intensity) as well as mitochondrial integrity (absorbance at 540 nm) was detected over time.

Representative data from at least three independent experiments.

Supplemental Figure 2: Vector map of Ad-CMV-mitoRL.

pENTR vector with expression cassette consisting of CMV promoter, dsRed linked to mitochondrial targeting site and luciferase gene linked by P2A sequences and followed by an bGH polyadenylation signal.

Supplemental Figure 3: Change of membrane potential after mixing of LCMV-infected and uninfected mitochondria

Flow cytometric analysis of membrane potential by $DilC_1(5)$ staining of mitochondria from uninfected livers with mitochondria from LCMV infected livers at a ratio 1:1. Samples were mixed before staining procedure (measurement 1h post mixing) .

Representative data from three independent experiments.

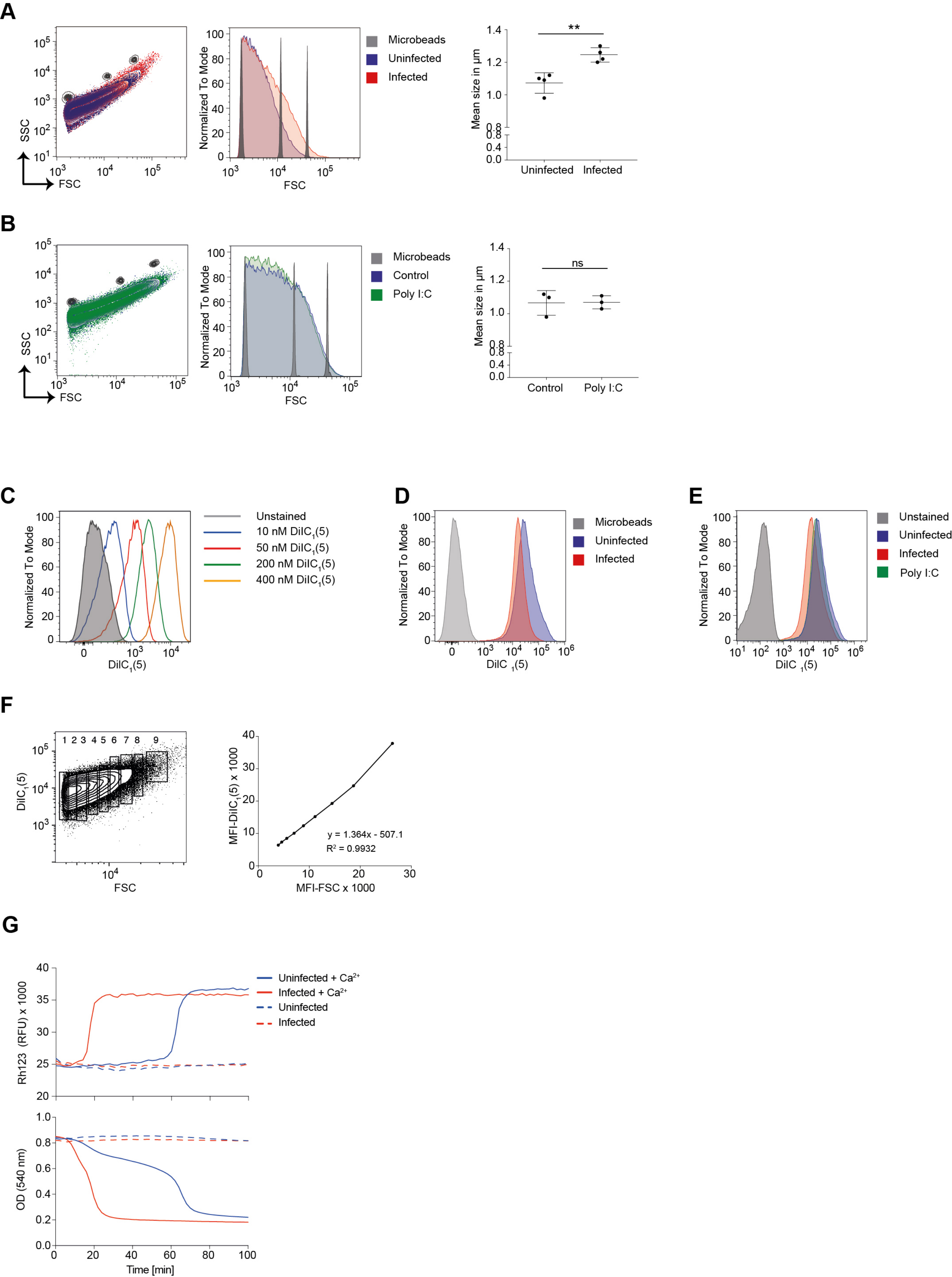
A
SSC
FSC
Normalized To Mode
Microbeads
Uninfected
Infected
Mean size in µm
**
B
Microbeads
Control
Poly I:C
ns
C
DilC1(5)
Unstained
10 nM DilC1(5)
50 nM DilC1(5)
200 nM DilC1(5)
400 nM DilC1(5)
D
E
F
1 2 3 4 5 6 7 8 9
MFI-DilC1(5) x 1000
MFI-FSC x 1000
y = 1.364x - 507.1
R² = 0.9932
G
Rh123 (RFU) x 1000
Uninfected + Ca2+
Infected + Ca2+
Uninfected
Infected
OD (540 nm)
Time [min]

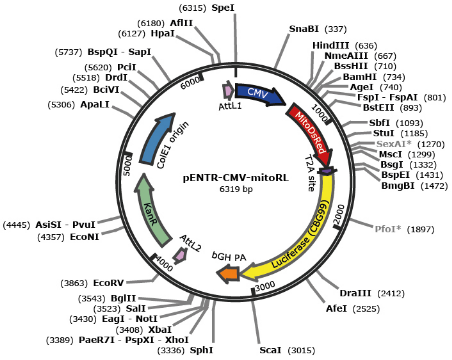

pENTR-CMV-mitoRL
6319 bp
(6315) SpeI
SnaBI (337)
HindIII (636)
NmeAIII (667)
BssHII (710)
BamHI (734)
AgeI (740)
FspI - FspAI (801)
BstEII (893)
SbfI (1093)
StuI (1185)
SexAI* (1270)
MscI (1299)
BsgI (1332)
BspEI (1431)
BmgBI (1472)
PfoI* (1897)
DraIII (2412)
AfeI (2525)
ScaI (3015)
(3336) SphI
(3389) PaeR7I - PspXI - XhoI
(3408) XbaI
(3430) EagI - NotI
(3523) SalI
(3543) BglII
(3863) EcoRV
(4357) EcoNI
(4445) AsiSI - PvuI
(5306) ApaLI
(5422) BciVI
(5518) DrdI
(5620) PciI
(5737) BspQI - SapI
(6127) HpaI
(6180) AflII
AttL1
CMV
MitoDsRed
T2A site
Luciferase (CBG99)
bGH PA
AttL2
KanR
ColE1 origin
1000
2000
3000
4000
5000
6000

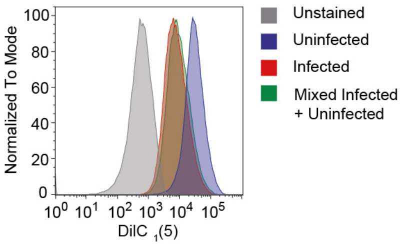

Normalized To Mode
100
80
60
40
20
0
10^0 10^1 10^2 10^3 10^4 10^5
DiIC 1(5)
Unstained
Uninfected
Infected
Mixed Infected
+ Uninfected
